# Poly[[(μ_2_-benzene-1,3-di­carboxyl­ato){μ_2_-1,4-bis­[(1*H*-imidazol-1-yl)meth­yl]benzene}­cadmium] di­methyl­formamide monosolvate]

**DOI:** 10.1107/S1600536813028298

**Published:** 2013-10-23

**Authors:** Yan Chen, Daguang Wang, Liang He, Wei Li, Jian Suo

**Affiliations:** a Department of General Surgery, The First Hospital, Jilin University, Changchun, 130021 Jilin Province, People’s Republic of China

## Abstract

The title coordination polymer, {[Cd(C_8_H_4_O_4_)(C_14_H_14_N_4_)]·C_3_H_7_NO}_*n*_, was synthesized by solvothermal reaction of metallic cadmium with the semi-rigid neutral ligand 1,4-bis­[(1*H*-imidazol-1-yl)meth­yl]benzene (bix) and the V-shaped benzene-1,3-di­carb­oxy­lic acid (*m*-H_2_bdc). The structure exhibits a pseudo-*C*-centring which is almost fulfilled by the polymeric metal complex but not by the solvent dimethylform­amide (DMF) mol­ecules. The asymmetric unit contains two independent Cd^II^ ions, two *m*-bdc^2−^ ligands, one and two half bix ligands, and two solvent DMF mol­ecules. The Cd^II^ ions are both five-coordinated by three O atoms from two different *m*-bdc^2−^ ligands and two N atoms from two different bix ligands in a distorted square-pyramidal geometry. The *m*-bdc^2−^ ligands adopt a chelate-monodentate coordination mode, connecting neighboring Cd^II^ ions into a zigzag chain parallel to [110]. Adjacent chains are further cross-linked by bix ligands, giving rise to a puckered sheet nearly perpendicular to the chain direction. Thus, each Cd^II^ ion is connected to four neighboring Cd^II^ ions through two *m*-bdc^2−^ anions and two bix ligands, giving rise to the final non-inter­penetrating uninodal layer with sql (4,4) topology.

## Related literature

For background compounds with metal-organic framework structurs, see: Batten & Robson (1998 ▶); Chen *et al.* (2011 ▶); Farrusseng *et al.* (2009 ▶); Kurmoo (2009 ▶); Pramanik *et al.* (2011 ▶); Wong *et al.* (2006 ▶). For topologies, see: Blatov *et al.* (2010 ▶). For a description of the geometry of complexes with five-coordinate metal atoms, see: Addison *et al.* (1984 ▶). 
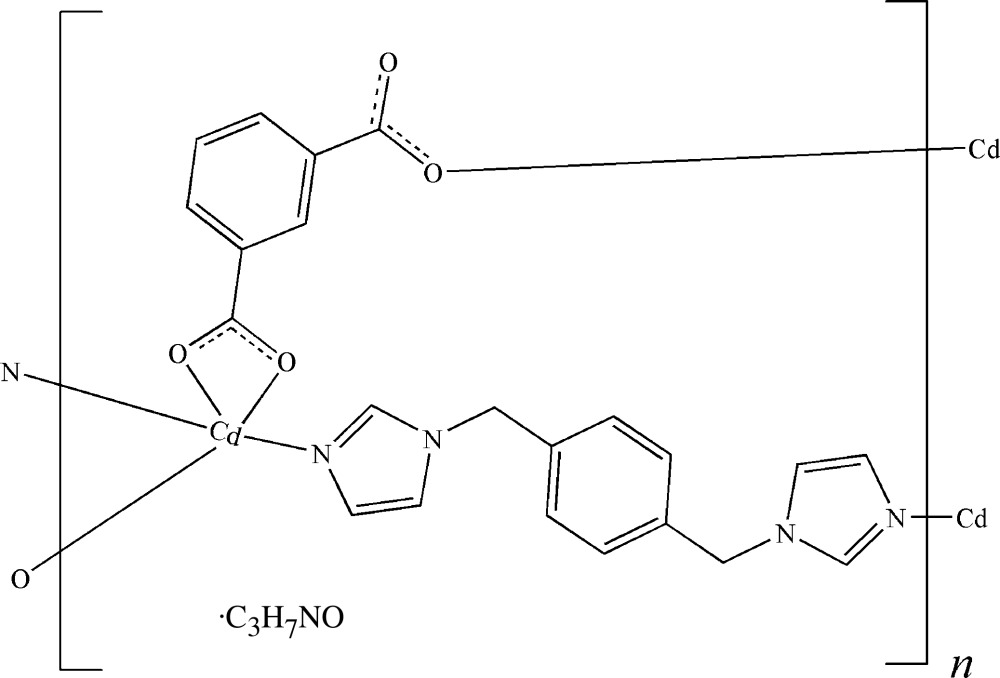


## Experimental

### 

#### Crystal data

[Cd(C_8_H_4_O_4_)(C_14_H_14_N_4_)]·C_3_H_7_NO
*M* *_r_* = 587.90
Triclinic, 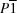

*a* = 11.2088 (4) Å
*b* = 13.4710 (5) Å
*c* = 18.9133 (7) Å
α = 69.648 (1)°
β = 80.124 (1)°
γ = 68.521 (1)°
*V* = 2488.37 (16) Å^3^
*Z* = 4
Mo *K*α radiation
μ = 0.92 mm^−1^
*T* = 185 K
0.26 × 0.23 × 0.13 mm

#### Data collection

Bruker APEXII CCD diffractometer
Absorption correction: multi-scan (*SADABS*; Bruker, 2007 ▶) *T*_min_ = 0.795, *T*_max_ = 0.889
13952 measured reflections
9746 independent reflections
6383 reflections with *I* > 2σ(*I*)
*R*_int_ = 0.017

#### Refinement

*R*[*F* ^2^ > 2σ(*F* ^2^)] = 0.034
*wR*(*F* ^2^) = 0.095
*S* = 1.05
9746 reflections
649 parameters
H-atom parameters constrained
Δρ_max_ = 0.61 e Å^−3^
Δρ_min_ = −0.51 e Å^−3^

### 

Data collection: *APEX2* (Bruker, 2007 ▶); cell refinement: *SAINT* (Bruker, 2007 ▶); data reduction: *SAINT*; program(s) used to solve structure: *SHELXTL* (Sheldrick, 2008 ▶); program(s) used to refine structure: *SHELXTL*; molecular graphics: *XP* in *SHELXTL* and *DIAMOND* (Brandenburg, 1999 ▶); software used to prepare material for publication: *SHELXTL*.

## Supplementary Material

Crystal structure: contains datablock(s) Suo1, I. DOI: 10.1107/S1600536813028298/bt6934sup1.cif

Structure factors: contains datablock(s) I. DOI: 10.1107/S1600536813028298/bt6934Isup2.hkl

Additional supplementary materials:  crystallographic information; 3D view; checkCIF report

## Figures and Tables

**Fig. 1.**
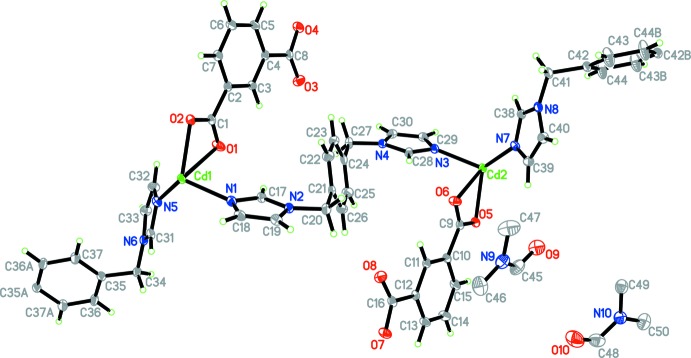
The asymmetric unit of the compound. Displacement ellipsoids are drawn at the 30% probability level. Symmetry codes: A = 2 - x,2 - y,-z; B = 1 - x,-1 - y,2 - z.

**Fig. 2.**
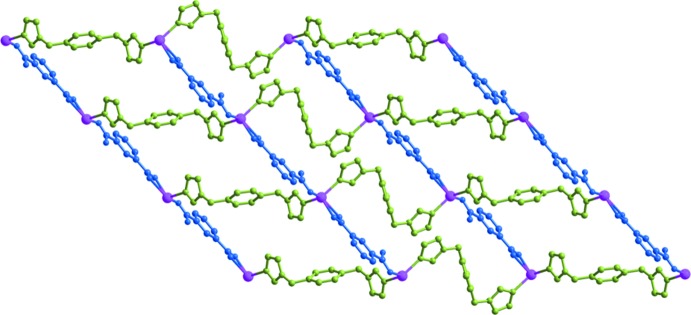
View of the single layer with the (4,4)-topology.

## supplementary crystallographic information

### 1. Comment

Studies of metal-organic coordination polymers (MOCPs) are of considerable interest due to their intrinsically interesting structures and fascinating network topologies (Batten *et al.*, 1998), and potential applications in storage (Chen *et al.*, 2011), catalysis (Farrusseng *et al.*, 2009), molecular magnetism (Kurmoo, 2009), recognition (Pramanik *et al.*, 2011), and photoluminescence (Wong *et al.*, 2006). An important motivation for this field is the rational design and preparation of crystalline solid material with peculiar topology and desired functions. The design possibilities of organic ligands and the coordination tendencies of metal ions have led to a large number of novel structural features quite often endowed with unique properties. Aromatic multi-acids are good connectors in constructed excellent porous coordination polymers due to their rigidity in conformation and various coordination modes. In particular, the combination of multicarboxylate anions with N-donor auxiliary ligands is a good choice for the construction of novel topology and networks.

In the present case, we used the benzene-1,3-dicarboxylic acid (*m*-H_2_bdc) as anionic ligand and 1,4-bis(imidazol-l-yl-methyl)benzene (bix) as ancillary ligand to construct a novel two-dimensional coordination polymer.

The structure shows pseudo-symmetry, in which the atoms in the main framework fulfill the pseudo-C centring and the DMF molecules break this pseudo-symmetry. The asymmetric unit contains two crystallographically independent Cd(II) ions, two *m*-bdc^2-^ ligands, one and two halves bix ligands, and two lattice DMF molecules (Fig. 1). Cd1 and Cd2 have the same coordination environment. They are five-coordinated by three oxygen atoms from two different *m*-bdc^2-^ ligands and two nitrogen atoms from two different bix ligands. The distortion parameters *τ*_5_ of 0.335 for both Cd1 and Cd2 indicate that the coordination environment corresponds to a distorted square pyramid; expected values are *τ* = 0 for a square pyramid and *τ* = 1 for an ideal trigonal bipyramid (Addison *et al.*, 1984).

As shown in Fig. 2, The *m*-bdc^2-^ ligands adopt a chelate-monodentate coordination mode, connect neighboring Cd ions into a zigzag chain. Adjacent chains are further cross-linked by bix ligands, giving rise to a 2-D puckered sheet with intragrid Cd···Cd separations of 10.245 (5) × 12.979 (5) Å and 10.193 (5) × 15.336 (5) Å across each *m*-bdc^2-^ ligand and bix ligand in the *trans*-conformation, respectively. Thus, the each Cd(II) is connected to four neighboring Cd atoms through two *m*-bdc^2-^ anions and two bix ligands to give rise to the final non-interpenetrating uninodal two-dimensional layer with sql topology (Blatov *et al.*, 2010). Furthermore, the adjacent two-dimensional layers stack in an ···AAA··· sequence, in which π-π interactions between the imidazol rings have a stabilizing effect [the cog(N1,N2,C17,C18,C19)···cog(N1,N2,C17,C18,C19)^i^ and cog(N3,N4,C28,C29,C30)···cog(N3,N4,C28,C29,C30)^ii^ distances are 3.517 Å and 3.478 Å, respectively; symmetry operators: (i) 2 - *x*,1 - *y*,1 - *z*, and (ii) 1 - *x*,-*y*,1 - *z*].

### 2. Experimental

A mixture of Cd(NO_3_)_2_·4H_2_O (0.0304 g, 0.1 mmol), *m*-H_2_bdc (0.0164 g, 0.1 mmol), bix (0.0238 g, 0.1 mmol), and DMF (6 ml) was placed in a 15 ml PTFE-lined stainless steel vessel under autogenous pressure, heated at a constant 95 °C for 72 h, and allowed to cool down to room temperature in 12 h. The title crystals were collected, washed with DMF and EtOH, and dried under ambient conditions with a yield of 13% based on Cd).

### 3. Refinement

All the hydrogen atoms attached to carbon atoms were placed in calculated positions and refined as the riding model.

### Crystal data

**Table:**

[Cd(C_8_H_4_O_4_)(C_14_H_14_N_4_)]·C_3_H_7_NO	*V* = 2488.37 (16) Å^3^
*M**_r_* = 587.90	*Z* = 4
Triclinic, *P*1	*F*(000) = 1192
Hall symbol: -P 1	*D*_x_ = 1.569 Mg m^−^^3^
*a* = 11.2088 (4) Å	Mo *K*α radiation, λ = 0.71073 Å
*b* = 13.4710 (5) Å	µ = 0.92 mm^−^^1^
*c* = 18.9133 (7) Å	*T* = 185 K
α = 69.648 (1)°	Needle, colorless
β = 80.124 (1)°	0.26 × 0.23 × 0.13 mm
γ = 68.521 (1)°	

### Data collection

**Table:**

Bruker APEXII CCD diffractometer	9746 independent reflections
Radiation source: fine-focus sealed tube	6383 reflections with *I* > 2σ(*I*)
Graphite monochromator	*R*_int_ = 0.017
φ and ω scans	θ_max_ = 26.1°, θ_min_ = 1.7°
Absorption correction: multi-scan (*SADABS*; Bruker, 2007)	*h* = −13→13
*T*_min_ = 0.795, *T*_max_ = 0.889	*k* = −10→16
13952 measured reflections	*l* = −21→23

### Refinement

**Table:**

Refinement on *F*^2^	Primary atom site location: structure-invariant direct methods
Least-squares matrix: full	Secondary atom site location: difference Fourier map
*R*[*F*^2^ > 2σ(*F*^2^)] = 0.034	Hydrogen site location: inferred from neighbouring sites
*wR*(*F*^2^) = 0.095	H-atom parameters constrained
*S* = 1.05	*w* = 1/[σ^2^(*F*_o_^2^) + (0.0431*P*)^2^ + 0.3924*P*] where *P* = (*F*_o_^2^ + 2*F*_c_^2^)/3
9746 reflections	(Δ/σ)_max_ = 0.010
649 parameters	Δρ_max_ = 0.61 e Å^−^^3^
0 restraints	Δρ_min_ = −0.51 e Å^−^^3^

### Special details

**Table:**

Geometry. All e.s.d.'s (except the e.s.d. in the dihedral angle between two l.s. planes) are estimated using the full covariance matrix. The cell e.s.d.'s are taken into account individually in the estimation of e.s.d.'s in distances, angles and torsion angles; correlations between e.s.d.'s in cell parameters are only used when they are defined by crystal symmetry. An approximate (isotropic) treatment of cell e.s.d.'s is used for estimating e.s.d.'s involving l.s. planes.
Refinement. Refinement of *F*^2^ against ALL reflections. The weighted *R*-factor *wR* and goodness of fit *S* are based on *F*^2^, conventional *R*-factors *R* are based on *F*, with *F* set to zero for negative *F*^2^. The threshold expression of *F*^2^ > σ(*F*^2^) is used only for calculating *R*-factors(gt) *etc*. and is not relevant to the choice of reflections for refinement. *R*-factors based on *F*^2^ are statistically about twice as large as those based on *F*, and *R*- factors based on ALL data will be even larger.

### Fractional atomic coordinates and isotropic or equivalent isotropic displacement parameters (Å^2^)

**Table:**

	*x*	*y*	*z*	*U*_iso_*/*U*_eq_	
C1	1.1683 (3)	0.2415 (3)	0.29177 (18)	0.0219 (7)	
C2	1.1610 (3)	0.1318 (3)	0.29320 (18)	0.0214 (7)	
C3	1.0430 (3)	0.1167 (3)	0.29977 (18)	0.0212 (7)	
H3	0.9666	0.1749	0.3063	0.025*	
C4	1.0352 (3)	0.0174 (3)	0.29695 (18)	0.0215 (7)	
C5	1.1478 (3)	−0.0674 (3)	0.28762 (19)	0.0268 (8)	
H5	1.1435	−0.1350	0.2846	0.032*	
C6	1.2658 (3)	−0.0539 (3)	0.2827 (2)	0.0285 (8)	
H6	1.3423	−0.1126	0.2771	0.034*	
C7	1.2727 (3)	0.0442 (3)	0.28584 (19)	0.0258 (8)	
H7	1.3541	0.0526	0.2830	0.031*	
C8	0.9071 (3)	0.0023 (3)	0.30248 (18)	0.0236 (8)	
C9	0.3346 (3)	0.2555 (3)	0.71091 (17)	0.0207 (7)	
C10	0.3391 (3)	0.3651 (3)	0.71219 (18)	0.0216 (7)	
C11	0.4556 (3)	0.3833 (3)	0.70514 (17)	0.0212 (7)	
H11	0.5331	0.3271	0.6972	0.025*	
C12	0.4597 (3)	0.4824 (3)	0.70960 (18)	0.0227 (7)	
C13	0.3459 (3)	0.5646 (3)	0.7210 (2)	0.0297 (8)	
H13	0.3482	0.6322	0.7251	0.036*	
C14	0.2295 (3)	0.5482 (3)	0.7265 (2)	0.0349 (9)	
H14	0.1518	0.6051	0.7333	0.042*	
C15	0.2262 (3)	0.4491 (3)	0.7220 (2)	0.0281 (8)	
H15	0.1460	0.4382	0.7257	0.034*	
C16	0.5861 (3)	0.5015 (3)	0.70207 (19)	0.0254 (8)	
C17	1.0306 (3)	0.3691 (3)	0.44554 (18)	0.0245 (8)	
H17	0.9744	0.3644	0.4154	0.029*	
C18	1.1861 (3)	0.3961 (3)	0.4807 (2)	0.0288 (8)	
H18	1.2608	0.4145	0.4792	0.035*	
C19	1.1161 (3)	0.3602 (3)	0.5442 (2)	0.0290 (8)	
H19	1.1322	0.3489	0.5946	0.035*	
C20	0.9167 (3)	0.3069 (3)	0.57294 (19)	0.0301 (8)	
H20A	0.8631	0.3673	0.5946	0.036*	
H20B	0.9574	0.2399	0.6151	0.036*	
C21	0.8322 (3)	0.2789 (3)	0.53413 (19)	0.0272 (8)	
C22	0.8780 (3)	0.1888 (3)	0.5069 (2)	0.0352 (9)	
H22	0.9659	0.1436	0.5116	0.042*	
C23	0.7991 (3)	0.1625 (3)	0.4728 (2)	0.0333 (9)	
H23	0.8334	0.1003	0.4539	0.040*	
C24	0.6703 (3)	0.2262 (3)	0.46594 (19)	0.0285 (8)	
C25	0.6239 (3)	0.3168 (3)	0.4934 (2)	0.0366 (9)	
H25	0.5359	0.3618	0.4891	0.044*	
C26	0.7035 (3)	0.3430 (3)	0.5270 (2)	0.0342 (9)	
H26	0.6697	0.4056	0.5454	0.041*	
C27	0.5851 (3)	0.1979 (3)	0.42727 (19)	0.0317 (9)	
H27A	0.5425	0.2654	0.3860	0.038*	
H27B	0.6390	0.1390	0.4044	0.038*	
C28	0.4746 (3)	0.1321 (3)	0.55469 (19)	0.0246 (8)	
H28	0.5299	0.1384	0.5846	0.030*	
C29	0.3222 (3)	0.0997 (3)	0.52056 (19)	0.0268 (8)	
H29	0.2493	0.0787	0.5224	0.032*	
C30	0.3898 (3)	0.1378 (3)	0.4571 (2)	0.0271 (8)	
H30	0.3737	0.1482	0.4068	0.032*	
C31	1.0636 (3)	0.6731 (3)	0.20771 (18)	0.0257 (8)	
H31	1.0874	0.6998	0.2417	0.031*	
C32	1.0492 (3)	0.5644 (3)	0.1510 (2)	0.0318 (9)	
H32	1.0618	0.4994	0.1377	0.038*	
C33	0.9706 (3)	0.6695 (3)	0.1168 (2)	0.0315 (9)	
H33	0.9187	0.6914	0.0756	0.038*	
C34	0.9112 (3)	0.8574 (3)	0.13767 (19)	0.0286 (8)	
H34A	0.9193	0.8803	0.1805	0.034*	
H34B	0.8190	0.8714	0.1341	0.034*	
C35	0.9595 (3)	0.9290 (3)	0.06538 (19)	0.0248 (8)	
C36	0.8811 (3)	1.0373 (3)	0.0336 (2)	0.0331 (9)	
H36	0.7984	1.0642	0.0564	0.040*	
C44	0.4296 (4)	−0.4321 (4)	0.9400 (2)	0.0576 (14)	
H44	0.3780	−0.3844	0.8982	0.069*	
C42	0.5491 (3)	−0.4309 (3)	0.93834 (19)	0.0279 (8)	
C43	0.6209 (4)	−0.4991 (4)	0.9993 (2)	0.0574 (14)	
H43	0.7055	−0.4995	1.0001	0.069*	
C37	1.0787 (3)	0.8924 (3)	0.0312 (2)	0.0343 (9)	
H37	1.1342	0.8180	0.0522	0.041*	
C41	0.6045 (3)	−0.3580 (3)	0.8699 (2)	0.0335 (9)	
H41A	0.6931	−0.3683	0.8795	0.040*	
H41B	0.6089	−0.3825	0.8257	0.040*	
C38	0.4515 (3)	−0.1767 (3)	0.79481 (18)	0.0253 (8)	
H38	0.4364	−0.2054	0.7591	0.030*	
C39	0.4469 (3)	−0.0653 (3)	0.85416 (19)	0.0290 (8)	
H39	0.4269	0.0008	0.8679	0.035*	
C40	0.5253 (3)	−0.1676 (3)	0.8912 (2)	0.0305 (9)	
H40	0.5702	−0.1870	0.9350	0.037*	
C45	0.3929 (4)	0.2343 (4)	0.9062 (2)	0.0527 (12)	
H45	0.3385	0.3102	0.8972	0.063*	
C46	0.5744 (5)	0.2978 (4)	0.8900 (3)	0.0687 (15)	
H46A	0.6680	0.2630	0.8904	0.103*	
H46B	0.5440	0.3341	0.9295	0.103*	
H46C	0.5506	0.3540	0.8407	0.103*	
C47	0.6051 (4)	0.0993 (4)	0.9156 (3)	0.0788 (17)	
H47A	0.6937	0.0985	0.9119	0.118*	
H47B	0.5939	0.0702	0.8773	0.118*	
H47C	0.5866	0.0521	0.9660	0.118*	
C48	0.0501 (5)	0.3563 (4)	1.0609 (3)	0.0595 (13)	
H48	−0.0195	0.4234	1.0597	0.071*	
C49	0.1270 (4)	0.1555 (3)	1.0914 (2)	0.0478 (11)	
H49A	0.0925	0.0936	1.1143	0.072*	
H49B	0.1965	0.1450	1.1212	0.072*	
H49C	0.1602	0.1568	1.0398	0.072*	
C50	−0.1017 (4)	0.2587 (4)	1.1177 (3)	0.0600 (14)	
H50A	−0.1005	0.1808	1.1365	0.090*	
H50B	−0.1611	0.3016	1.0768	0.090*	
H50C	−0.1297	0.2919	1.1589	0.090*	
N1	1.1316 (2)	0.4017 (2)	0.41875 (15)	0.0253 (7)	
N2	1.0180 (3)	0.3435 (2)	0.52109 (15)	0.0235 (6)	
N3	0.3755 (2)	0.0960 (2)	0.58225 (15)	0.0244 (6)	
N4	0.4866 (2)	0.1584 (2)	0.47926 (15)	0.0227 (6)	
N5	1.1076 (3)	0.5675 (2)	0.20816 (15)	0.0261 (7)	
N6	0.9807 (3)	0.7379 (2)	0.15290 (15)	0.0242 (6)	
N7	0.4000 (2)	−0.0710 (2)	0.79320 (15)	0.0245 (6)	
N8	0.5273 (3)	−0.2384 (2)	0.85267 (16)	0.0260 (7)	
N9	0.5173 (3)	0.2141 (3)	0.90346 (19)	0.0452 (9)	
N10	0.0256 (3)	0.2612 (3)	1.08964 (18)	0.0417 (8)	
O1	1.0681 (2)	0.32236 (19)	0.29137 (15)	0.0343 (6)	
O2	1.2794 (2)	0.24650 (18)	0.29156 (13)	0.0255 (5)	
O3	0.8062 (2)	0.0766 (2)	0.31280 (15)	0.0368 (6)	
O4	0.9086 (2)	−0.0881 (2)	0.29473 (14)	0.0314 (6)	
O5	0.2247 (2)	0.24750 (18)	0.71208 (12)	0.0250 (5)	
O6	0.4361 (2)	0.1771 (2)	0.70900 (14)	0.0316 (6)	
O7	0.5820 (2)	0.5907 (2)	0.71313 (14)	0.0328 (6)	
O8	0.6875 (2)	0.4331 (2)	0.68585 (15)	0.0365 (6)	
O9	0.3411 (3)	0.1626 (3)	0.91959 (18)	0.0579 (9)	
O10	0.1555 (3)	0.3657 (3)	1.0354 (2)	0.0789 (11)	
Cd1	1.21121 (2)	0.423270 (19)	0.301290 (13)	0.02138 (8)	
Cd2	0.29640 (2)	0.072572 (19)	0.699760 (13)	0.02152 (8)	

### Atomic displacement parameters (Å^2^)

**Table:**

	*U*^11^	*U*^22^	*U*^33^	*U*^12^	*U*^13^	*U*^23^
C1	0.0223 (17)	0.026 (2)	0.0197 (18)	−0.0142 (15)	−0.0018 (14)	−0.0031 (15)
C2	0.0220 (16)	0.0245 (19)	0.0208 (18)	−0.0121 (15)	0.0003 (13)	−0.0068 (15)
C3	0.0191 (16)	0.0215 (18)	0.0244 (19)	−0.0094 (14)	−0.0017 (13)	−0.0055 (15)
C4	0.0194 (16)	0.0251 (19)	0.0222 (18)	−0.0111 (15)	0.0014 (13)	−0.0072 (15)
C5	0.0295 (18)	0.0205 (19)	0.034 (2)	−0.0130 (15)	−0.0003 (15)	−0.0077 (16)
C6	0.0191 (16)	0.0220 (19)	0.043 (2)	−0.0065 (15)	−0.0037 (15)	−0.0080 (17)
C7	0.0177 (16)	0.029 (2)	0.033 (2)	−0.0121 (15)	−0.0012 (14)	−0.0088 (16)
C8	0.0270 (18)	0.030 (2)	0.0190 (18)	−0.0182 (16)	0.0032 (14)	−0.0064 (15)
C9	0.0217 (16)	0.0240 (19)	0.0189 (18)	−0.0119 (15)	−0.0019 (13)	−0.0045 (15)
C10	0.0221 (16)	0.0220 (19)	0.0222 (18)	−0.0107 (15)	−0.0035 (14)	−0.0041 (15)
C11	0.0179 (15)	0.0257 (19)	0.0217 (18)	−0.0093 (14)	0.0003 (13)	−0.0079 (15)
C12	0.0197 (16)	0.0249 (19)	0.0239 (19)	−0.0121 (15)	0.0005 (14)	−0.0039 (15)
C13	0.0261 (18)	0.026 (2)	0.042 (2)	−0.0121 (16)	0.0000 (16)	−0.0130 (17)
C14	0.0188 (17)	0.029 (2)	0.058 (3)	−0.0078 (16)	0.0010 (17)	−0.0167 (19)
C15	0.0168 (16)	0.029 (2)	0.041 (2)	−0.0121 (15)	−0.0029 (15)	−0.0090 (17)
C16	0.0273 (18)	0.029 (2)	0.026 (2)	−0.0181 (16)	−0.0037 (15)	−0.0058 (16)
C17	0.0261 (17)	0.0254 (19)	0.0215 (19)	−0.0107 (15)	−0.0012 (14)	−0.0045 (15)
C18	0.0263 (18)	0.024 (2)	0.038 (2)	−0.0096 (16)	−0.0056 (16)	−0.0086 (17)
C19	0.0307 (19)	0.028 (2)	0.030 (2)	−0.0097 (16)	−0.0062 (16)	−0.0080 (16)
C20	0.0312 (19)	0.032 (2)	0.027 (2)	−0.0144 (17)	0.0033 (16)	−0.0067 (17)
C21	0.0291 (18)	0.028 (2)	0.024 (2)	−0.0163 (16)	0.0077 (15)	−0.0041 (16)
C22	0.0249 (18)	0.034 (2)	0.046 (2)	−0.0075 (17)	0.0018 (17)	−0.0155 (19)
C23	0.034 (2)	0.031 (2)	0.037 (2)	−0.0138 (17)	0.0087 (17)	−0.0142 (17)
C24	0.0301 (19)	0.029 (2)	0.023 (2)	−0.0164 (16)	0.0061 (15)	−0.0005 (16)
C25	0.0242 (18)	0.033 (2)	0.050 (3)	−0.0064 (17)	0.0024 (17)	−0.0140 (19)
C26	0.032 (2)	0.029 (2)	0.045 (2)	−0.0126 (17)	0.0062 (17)	−0.0173 (18)
C27	0.0340 (19)	0.035 (2)	0.023 (2)	−0.0162 (17)	0.0038 (16)	−0.0034 (17)
C28	0.0204 (16)	0.027 (2)	0.0252 (19)	−0.0088 (15)	0.0015 (14)	−0.0076 (16)
C29	0.0235 (17)	0.027 (2)	0.034 (2)	−0.0091 (15)	−0.0046 (15)	−0.0111 (17)
C30	0.0290 (18)	0.027 (2)	0.025 (2)	−0.0055 (16)	−0.0073 (15)	−0.0100 (16)
C31	0.0281 (18)	0.026 (2)	0.0229 (19)	−0.0145 (16)	0.0007 (15)	−0.0029 (15)
C32	0.039 (2)	0.029 (2)	0.030 (2)	−0.0107 (17)	−0.0066 (17)	−0.0114 (17)
C33	0.036 (2)	0.034 (2)	0.027 (2)	−0.0106 (18)	−0.0063 (16)	−0.0111 (17)
C34	0.0302 (18)	0.022 (2)	0.027 (2)	−0.0065 (16)	0.0055 (15)	−0.0053 (16)
C35	0.0262 (17)	0.0218 (19)	0.0247 (19)	−0.0089 (15)	−0.0005 (14)	−0.0047 (15)
C36	0.0239 (17)	0.031 (2)	0.034 (2)	−0.0072 (16)	0.0075 (15)	−0.0040 (17)
C44	0.035 (2)	0.066 (3)	0.044 (3)	−0.020 (2)	−0.011 (2)	0.023 (2)
C42	0.0269 (18)	0.0216 (19)	0.027 (2)	−0.0051 (15)	0.0029 (15)	−0.0026 (16)
C43	0.033 (2)	0.062 (3)	0.055 (3)	−0.021 (2)	−0.008 (2)	0.016 (2)
C37	0.0257 (18)	0.027 (2)	0.036 (2)	−0.0026 (16)	0.0013 (16)	−0.0007 (17)
C41	0.0301 (19)	0.024 (2)	0.033 (2)	−0.0061 (16)	0.0084 (16)	−0.0009 (17)
C38	0.0317 (18)	0.025 (2)	0.0220 (19)	−0.0141 (16)	0.0008 (15)	−0.0067 (15)
C39	0.0324 (19)	0.028 (2)	0.030 (2)	−0.0119 (17)	−0.0014 (16)	−0.0117 (17)
C40	0.0300 (19)	0.033 (2)	0.026 (2)	−0.0068 (17)	−0.0057 (16)	−0.0075 (17)
C45	0.046 (3)	0.072 (4)	0.039 (3)	−0.010 (3)	−0.007 (2)	−0.024 (2)
C46	0.066 (3)	0.077 (4)	0.061 (3)	−0.031 (3)	−0.012 (3)	−0.007 (3)
C47	0.049 (3)	0.088 (4)	0.115 (5)	−0.012 (3)	−0.012 (3)	−0.060 (4)
C48	0.068 (3)	0.051 (3)	0.066 (3)	−0.016 (3)	−0.016 (3)	−0.022 (3)
C49	0.045 (2)	0.050 (3)	0.041 (3)	−0.012 (2)	−0.005 (2)	−0.008 (2)
C50	0.049 (3)	0.071 (4)	0.049 (3)	−0.013 (3)	−0.004 (2)	−0.013 (3)
N1	0.0246 (14)	0.0242 (16)	0.0280 (17)	−0.0105 (13)	−0.0013 (12)	−0.0067 (13)
N2	0.0244 (14)	0.0217 (16)	0.0226 (16)	−0.0079 (12)	−0.0001 (12)	−0.0050 (13)
N3	0.0230 (14)	0.0234 (16)	0.0265 (16)	−0.0099 (12)	0.0024 (12)	−0.0069 (13)
N4	0.0221 (14)	0.0207 (16)	0.0233 (16)	−0.0067 (12)	0.0012 (12)	−0.0061 (13)
N5	0.0268 (15)	0.0236 (17)	0.0269 (17)	−0.0103 (13)	−0.0033 (12)	−0.0038 (13)
N6	0.0263 (15)	0.0243 (16)	0.0192 (15)	−0.0102 (13)	0.0019 (12)	−0.0030 (13)
N7	0.0251 (14)	0.0218 (16)	0.0274 (17)	−0.0114 (13)	−0.0030 (12)	−0.0040 (13)
N8	0.0242 (14)	0.0223 (16)	0.0251 (17)	−0.0068 (13)	0.0021 (12)	−0.0024 (13)
N9	0.044 (2)	0.057 (3)	0.038 (2)	−0.0153 (19)	−0.0092 (16)	−0.0175 (18)
N10	0.042 (2)	0.039 (2)	0.039 (2)	−0.0082 (17)	−0.0100 (16)	−0.0088 (16)
O1	0.0246 (13)	0.0236 (14)	0.0580 (18)	−0.0090 (11)	−0.0105 (12)	−0.0115 (12)
O2	0.0227 (12)	0.0281 (14)	0.0323 (14)	−0.0154 (10)	0.0022 (10)	−0.0115 (11)
O3	0.0217 (13)	0.0466 (17)	0.0551 (18)	−0.0165 (12)	0.0079 (12)	−0.0305 (14)
O4	0.0252 (13)	0.0307 (15)	0.0475 (17)	−0.0179 (11)	−0.0027 (11)	−0.0134 (12)
O5	0.0222 (11)	0.0271 (14)	0.0309 (14)	−0.0155 (10)	0.0016 (10)	−0.0090 (11)
O6	0.0239 (13)	0.0276 (14)	0.0487 (17)	−0.0089 (11)	−0.0049 (11)	−0.0164 (12)
O7	0.0269 (13)	0.0288 (15)	0.0505 (17)	−0.0145 (11)	−0.0046 (11)	−0.0149 (12)
O8	0.0224 (13)	0.0459 (17)	0.0522 (18)	−0.0181 (12)	0.0090 (12)	−0.0265 (14)
O9	0.0438 (18)	0.064 (2)	0.070 (2)	−0.0162 (16)	−0.0054 (16)	−0.0264 (18)
O10	0.066 (2)	0.073 (3)	0.113 (3)	−0.041 (2)	0.001 (2)	−0.031 (2)
Cd1	0.01834 (13)	0.02126 (15)	0.02570 (16)	−0.00977 (11)	0.00102 (10)	−0.00628 (11)
Cd2	0.01866 (13)	0.02158 (15)	0.02509 (16)	−0.00998 (11)	0.00115 (10)	−0.00570 (11)

### Geometric parameters (Å, º)

**Table:**

C1—O1	1.244 (4)	C31—N6	1.344 (4)
C1—O2	1.271 (4)	C31—H31	0.9500
C1—C2	1.499 (4)	C32—C33	1.358 (5)
C1—Cd1	2.731 (3)	C32—N5	1.376 (4)
C2—C3	1.390 (4)	C32—H32	0.9500
C2—C7	1.397 (4)	C33—N6	1.367 (4)
C3—C4	1.391 (4)	C33—H33	0.9500
C3—H3	0.9500	C34—N6	1.456 (4)
C4—C5	1.391 (4)	C34—C35	1.518 (4)
C4—C8	1.504 (4)	C34—H34A	0.9900
C5—C6	1.384 (4)	C34—H34B	0.9900
C5—H5	0.9500	C35—C37	1.374 (5)
C6—C7	1.374 (4)	C35—C36	1.377 (4)
C6—H6	0.9500	C36—C37^i^	1.384 (5)
C7—H7	0.9500	C36—H36	0.9500
C8—O3	1.245 (4)	C44—C42	1.341 (5)
C8—O4	1.270 (4)	C44—C43^ii^	1.384 (5)
C9—O6	1.243 (4)	C44—H44	0.9500
C9—O5	1.270 (4)	C42—C43	1.364 (5)
C9—C10	1.505 (4)	C42—C41	1.520 (4)
C9—Cd2	2.732 (3)	C43—C44^ii^	1.384 (5)
C10—C15	1.390 (4)	C43—H43	0.9500
C10—C11	1.391 (4)	C37—C36^i^	1.384 (5)
C11—C12	1.385 (4)	C37—H37	0.9500
C11—H11	0.9500	C41—N8	1.469 (4)
C12—C13	1.392 (4)	C41—H41A	0.9900
C12—C16	1.506 (4)	C41—H41B	0.9900
C13—C14	1.382 (5)	C38—N7	1.317 (4)
C13—H13	0.9500	C38—N8	1.334 (4)
C14—C15	1.382 (5)	C38—H38	0.9500
C14—H14	0.9500	C39—C40	1.347 (5)
C15—H15	0.9500	C39—N7	1.383 (4)
C16—O8	1.243 (4)	C39—H39	0.9500
C16—O7	1.273 (4)	C40—N8	1.380 (4)
C17—N1	1.321 (4)	C40—H40	0.9500
C17—N2	1.343 (4)	C45—O9	1.235 (5)
C17—H17	0.9500	C45—N9	1.315 (5)
C18—C19	1.357 (5)	C45—H45	0.9500
C18—N1	1.379 (4)	C46—N9	1.422 (5)
C18—H18	0.9500	C46—H46A	0.9800
C19—N2	1.365 (4)	C46—H46B	0.9800
C19—H19	0.9500	C46—H46C	0.9800
C20—N2	1.479 (4)	C47—N9	1.458 (5)
C20—C21	1.507 (5)	C47—H47A	0.9800
C20—H20A	0.9900	C47—H47B	0.9800
C20—H20B	0.9900	C47—H47C	0.9800
C21—C22	1.375 (5)	C48—O10	1.231 (6)
C21—C26	1.384 (5)	C48—N10	1.317 (5)
C22—C23	1.381 (5)	C48—H48	0.9500
C22—H22	0.9500	C49—N10	1.455 (5)
C23—C24	1.384 (5)	C49—H49A	0.9800
C23—H23	0.9500	C49—H49B	0.9800
C24—C25	1.384 (5)	C49—H49C	0.9800
C24—C27	1.514 (5)	C50—N10	1.442 (5)
C25—C26	1.380 (5)	C50—H50A	0.9800
C25—H25	0.9500	C50—H50B	0.9800
C26—H26	0.9500	C50—H50C	0.9800
C27—N4	1.471 (4)	N1—Cd1	2.208 (3)
C27—H27A	0.9900	N3—Cd2	2.206 (3)
C27—H27B	0.9900	N5—Cd1	2.213 (3)
C28—N3	1.328 (4)	N7—Cd2	2.212 (3)
C28—N4	1.340 (4)	O1—Cd1	2.517 (2)
C28—H28	0.9500	O2—Cd1	2.283 (2)
C29—C30	1.351 (5)	O4—Cd2^iii^	2.217 (2)
C29—N3	1.378 (4)	O5—Cd2	2.277 (2)
C29—H29	0.9500	O6—Cd2	2.519 (2)
C30—N4	1.374 (4)	O7—Cd1^iv^	2.232 (2)
C30—H30	0.9500	Cd1—O7^iv^	2.232 (2)
C31—N5	1.321 (4)	Cd2—O4^iii^	2.217 (2)
			
O1—C1—O2	122.9 (3)	C42—C44—C43^ii^	122.0 (4)
O1—C1—C2	120.1 (3)	C42—C44—H44	119.0
O2—C1—C2	117.1 (3)	C43^ii^—C44—H44	119.0
O1—C1—Cd1	66.82 (18)	C44—C42—C43	117.9 (3)
O2—C1—Cd1	56.19 (16)	C44—C42—C41	121.2 (3)
C2—C1—Cd1	171.8 (2)	C43—C42—C41	120.9 (3)
C3—C2—C7	118.8 (3)	C42—C43—C44^ii^	120.1 (4)
C3—C2—C1	120.6 (3)	C42—C43—H43	120.0
C7—C2—C1	120.5 (3)	C44^ii^—C43—H43	120.0
C2—C3—C4	120.9 (3)	C35—C37—C36^i^	120.9 (3)
C2—C3—H3	119.6	C35—C37—H37	119.6
C4—C3—H3	119.6	C36^i^—C37—H37	119.6
C3—C4—C5	119.1 (3)	N8—C41—C42	112.2 (3)
C3—C4—C8	120.7 (3)	N8—C41—H41A	109.2
C5—C4—C8	120.2 (3)	C42—C41—H41A	109.2
C6—C5—C4	120.4 (3)	N8—C41—H41B	109.2
C6—C5—H5	119.8	C42—C41—H41B	109.2
C4—C5—H5	119.8	H41A—C41—H41B	107.9
C7—C6—C5	120.2 (3)	N7—C38—N8	111.8 (3)
C7—C6—H6	119.9	N7—C38—H38	124.1
C5—C6—H6	119.9	N8—C38—H38	124.1
C6—C7—C2	120.6 (3)	C40—C39—N7	109.6 (3)
C6—C7—H7	119.7	C40—C39—H39	125.2
C2—C7—H7	119.7	N7—C39—H39	125.2
O3—C8—O4	122.9 (3)	C39—C40—N8	106.1 (3)
O3—C8—C4	120.6 (3)	C39—C40—H40	126.9
O4—C8—C4	116.4 (3)	N8—C40—H40	126.9
O6—C9—O5	122.7 (3)	O9—C45—N9	125.2 (5)
O6—C9—C10	119.8 (3)	O9—C45—H45	117.4
O5—C9—C10	117.5 (3)	N9—C45—H45	117.4
O6—C9—Cd2	66.90 (18)	N9—C46—H46A	109.5
O5—C9—Cd2	55.89 (16)	N9—C46—H46B	109.5
C10—C9—Cd2	172.5 (2)	H46A—C46—H46B	109.5
C15—C10—C11	119.1 (3)	N9—C46—H46C	109.5
C15—C10—C9	120.1 (3)	H46A—C46—H46C	109.5
C11—C10—C9	120.8 (3)	H46B—C46—H46C	109.5
C12—C11—C10	120.6 (3)	N9—C47—H47A	109.5
C12—C11—H11	119.7	N9—C47—H47B	109.5
C10—C11—H11	119.7	H47A—C47—H47B	109.5
C11—C12—C13	119.5 (3)	N9—C47—H47C	109.5
C11—C12—C16	120.4 (3)	H47A—C47—H47C	109.5
C13—C12—C16	120.1 (3)	H47B—C47—H47C	109.5
C14—C13—C12	120.2 (3)	O10—C48—N10	125.7 (5)
C14—C13—H13	119.9	O10—C48—H48	117.2
C12—C13—H13	119.9	N10—C48—H48	117.2
C15—C14—C13	119.9 (3)	N10—C49—H49A	109.5
C15—C14—H14	120.0	N10—C49—H49B	109.5
C13—C14—H14	120.0	H49A—C49—H49B	109.5
C14—C15—C10	120.6 (3)	N10—C49—H49C	109.5
C14—C15—H15	119.7	H49A—C49—H49C	109.5
C10—C15—H15	119.7	H49B—C49—H49C	109.5
O8—C16—O7	122.9 (3)	N10—C50—H50A	109.5
O8—C16—C12	120.9 (3)	N10—C50—H50B	109.5
O7—C16—C12	116.1 (3)	H50A—C50—H50B	109.5
N1—C17—N2	110.9 (3)	N10—C50—H50C	109.5
N1—C17—H17	124.6	H50A—C50—H50C	109.5
N2—C17—H17	124.6	H50B—C50—H50C	109.5
C19—C18—N1	109.3 (3)	C17—N1—C18	105.9 (3)
C19—C18—H18	125.4	C17—N1—Cd1	123.9 (2)
N1—C18—H18	125.4	C18—N1—Cd1	129.0 (2)
C18—C19—N2	106.3 (3)	C17—N2—C19	107.8 (3)
C18—C19—H19	126.9	C17—N2—C20	128.4 (3)
N2—C19—H19	126.9	C19—N2—C20	123.8 (3)
N2—C20—C21	112.5 (3)	C28—N3—C29	105.6 (3)
N2—C20—H20A	109.1	C28—N3—Cd2	124.2 (2)
C21—C20—H20A	109.1	C29—N3—Cd2	129.0 (2)
N2—C20—H20B	109.1	C28—N4—C30	107.1 (3)
C21—C20—H20B	109.1	C28—N4—C27	128.6 (3)
H20A—C20—H20B	107.8	C30—N4—C27	124.2 (3)
C22—C21—C26	118.1 (3)	C31—N5—C32	106.1 (3)
C22—C21—C20	121.9 (3)	C31—N5—Cd1	124.8 (2)
C26—C21—C20	119.9 (3)	C32—N5—Cd1	127.3 (2)
C21—C22—C23	121.5 (3)	C31—N6—C33	107.1 (3)
C21—C22—H22	119.3	C31—N6—C34	125.9 (3)
C23—C22—H22	119.3	C33—N6—C34	126.9 (3)
C22—C23—C24	120.4 (4)	C38—N7—C39	105.3 (3)
C22—C23—H23	119.8	C38—N7—Cd2	126.9 (2)
C24—C23—H23	119.8	C39—N7—Cd2	126.5 (2)
C25—C24—C23	118.2 (4)	C38—N8—C40	107.1 (3)
C25—C24—C27	121.7 (3)	C38—N8—C41	126.7 (3)
C23—C24—C27	120.0 (3)	C40—N8—C41	126.1 (3)
C26—C25—C24	121.0 (3)	C45—N9—C46	124.1 (4)
C26—C25—H25	119.5	C45—N9—C47	119.6 (4)
C24—C25—H25	119.5	C46—N9—C47	116.3 (4)
C25—C26—C21	120.7 (4)	C48—N10—C50	121.7 (4)
C25—C26—H26	119.6	C48—N10—C49	120.1 (4)
C21—C26—H26	119.6	C50—N10—C49	118.3 (4)
N4—C27—C24	112.7 (3)	C1—O1—Cd1	86.14 (19)
N4—C27—H27A	109.0	C1—O2—Cd1	96.3 (2)
C24—C27—H27A	109.0	C8—O4—Cd2^iii^	104.9 (2)
N4—C27—H27B	109.0	C9—O5—Cd2	96.6 (2)
C24—C27—H27B	109.0	C9—O6—Cd2	86.10 (19)
H27A—C27—H27B	107.8	C16—O7—Cd1^iv^	102.0 (2)
N3—C28—N4	111.2 (3)	N1—Cd1—N5	120.05 (10)
N3—C28—H28	124.4	N1—Cd1—O7^iv^	113.90 (9)
N4—C28—H28	124.4	N5—Cd1—O7^iv^	108.45 (9)
C30—C29—N3	109.4 (3)	N1—Cd1—O2	103.81 (9)
C30—C29—H29	125.3	N5—Cd1—O2	119.59 (9)
N3—C29—H29	125.3	O7^iv^—Cd1—O2	86.36 (8)
C29—C30—N4	106.6 (3)	N1—Cd1—O1	85.77 (9)
C29—C30—H30	126.7	N5—Cd1—O1	87.77 (9)
N4—C30—H30	126.7	O7^iv^—Cd1—O1	140.15 (8)
N5—C31—N6	111.1 (3)	O2—Cd1—O1	54.54 (7)
N5—C31—H31	124.4	N1—Cd1—C1	94.16 (10)
N6—C31—H31	124.4	N5—Cd1—C1	105.59 (10)
C33—C32—N5	108.9 (3)	O7^iv^—Cd1—C1	113.76 (9)
C33—C32—H32	125.6	O2—Cd1—C1	27.55 (8)
N5—C32—H32	125.6	O1—Cd1—C1	27.04 (8)
C32—C33—N6	106.8 (3)	N3—Cd2—N7	120.66 (10)
C32—C33—H33	126.6	N3—Cd2—O4^iii^	109.82 (9)
N6—C33—H33	126.6	N7—Cd2—O4^iii^	111.39 (9)
N6—C34—C35	113.0 (3)	N3—Cd2—O5	104.62 (9)
N6—C34—H34A	109.0	N7—Cd2—O5	118.63 (9)
C35—C34—H34A	109.0	O4^iii^—Cd2—O5	86.42 (8)
N6—C34—H34B	109.0	N3—Cd2—O6	85.43 (9)
C35—C34—H34B	109.0	N7—Cd2—O6	88.45 (9)
H34A—C34—H34B	107.8	O4^iii^—Cd2—O6	140.80 (8)
C37—C35—C36	118.3 (3)	O5—Cd2—O6	54.46 (7)
C37—C35—C34	123.5 (3)	N3—Cd2—C9	94.51 (10)
C36—C35—C34	118.2 (3)	N7—Cd2—C9	105.43 (10)
C35—C36—C37^i^	120.8 (3)	O4^iii^—Cd2—C9	113.93 (9)
C35—C36—H36	119.6	O5—Cd2—C9	27.51 (8)
C37^i^—C36—H36	119.6	O6—Cd2—C9	26.99 (8)
			
O1—C1—C2—C3	−4.8 (5)	N7—C38—N8—C40	−0.3 (4)
O2—C1—C2—C3	174.7 (3)	N7—C38—N8—C41	−177.1 (3)
Cd1—C1—C2—C3	141.5 (15)	C39—C40—N8—C38	0.2 (4)
O1—C1—C2—C7	173.4 (3)	C39—C40—N8—C41	177.0 (3)
O2—C1—C2—C7	−7.1 (5)	C42—C41—N8—C38	−107.6 (4)
Cd1—C1—C2—C7	−40.3 (18)	C42—C41—N8—C40	76.2 (4)
C7—C2—C3—C4	−1.8 (5)	O9—C45—N9—C46	−178.2 (4)
C1—C2—C3—C4	176.4 (3)	O9—C45—N9—C47	1.0 (7)
C2—C3—C4—C5	0.2 (5)	O10—C48—N10—C50	−179.5 (5)
C2—C3—C4—C8	−178.7 (3)	O10—C48—N10—C49	−1.6 (7)
C3—C4—C5—C6	1.2 (5)	O2—C1—O1—Cd1	−4.4 (3)
C8—C4—C5—C6	−179.9 (3)	C2—C1—O1—Cd1	175.1 (3)
C4—C5—C6—C7	−1.0 (5)	O1—C1—O2—Cd1	4.9 (3)
C5—C6—C7—C2	−0.7 (5)	C2—C1—O2—Cd1	−174.6 (2)
C3—C2—C7—C6	2.1 (5)	O3—C8—O4—Cd2^iii^	1.8 (4)
C1—C2—C7—C6	−176.1 (3)	C4—C8—O4—Cd2^iii^	−177.0 (2)
C3—C4—C8—O3	−3.0 (5)	O6—C9—O5—Cd2	−4.4 (3)
C5—C4—C8—O3	178.1 (3)	C10—C9—O5—Cd2	175.8 (2)
C3—C4—C8—O4	175.8 (3)	O5—C9—O6—Cd2	4.0 (3)
C5—C4—C8—O4	−3.1 (5)	C10—C9—O6—Cd2	−176.3 (3)
O6—C9—C10—C15	−173.5 (3)	O8—C16—O7—Cd1^iv^	−1.5 (4)
O5—C9—C10—C15	6.3 (5)	C12—C16—O7—Cd1^iv^	178.6 (2)
Cd2—C9—C10—C15	33.8 (19)	C17—N1—Cd1—N5	72.8 (3)
O6—C9—C10—C11	5.2 (5)	C18—N1—Cd1—N5	−121.5 (3)
O5—C9—C10—C11	−175.0 (3)	C17—N1—Cd1—O7^iv^	−156.2 (2)
Cd2—C9—C10—C11	−147.5 (16)	C18—N1—Cd1—O7^iv^	9.6 (3)
C15—C10—C11—C12	1.5 (5)	C17—N1—Cd1—O2	−64.0 (3)
C9—C10—C11—C12	−177.2 (3)	C18—N1—Cd1—O2	101.7 (3)
C10—C11—C12—C13	−0.2 (5)	C17—N1—Cd1—O1	−12.2 (3)
C10—C11—C12—C16	179.9 (3)	C18—N1—Cd1—O1	153.6 (3)
C11—C12—C13—C14	−1.2 (5)	C17—N1—Cd1—C1	−37.9 (3)
C16—C12—C13—C14	178.7 (3)	C18—N1—Cd1—C1	127.9 (3)
C12—C13—C14—C15	1.2 (6)	C31—N5—Cd1—N1	49.6 (3)
C13—C14—C15—C10	0.1 (6)	C32—N5—Cd1—N1	−112.7 (3)
C11—C10—C15—C14	−1.5 (5)	C31—N5—Cd1—O7^iv^	−83.7 (3)
C9—C10—C15—C14	177.3 (3)	C32—N5—Cd1—O7^iv^	113.9 (3)
C11—C12—C16—O8	5.7 (5)	C31—N5—Cd1—O2	179.8 (2)
C13—C12—C16—O8	−174.2 (3)	C32—N5—Cd1—O2	17.5 (3)
C11—C12—C16—O7	−174.4 (3)	C31—N5—Cd1—O1	133.4 (3)
C13—C12—C16—O7	5.7 (5)	C32—N5—Cd1—O1	−28.9 (3)
N1—C18—C19—N2	0.0 (4)	C31—N5—Cd1—C1	154.0 (2)
N2—C20—C21—C22	66.8 (4)	C32—N5—Cd1—C1	−8.3 (3)
N2—C20—C21—C26	−115.0 (4)	C1—O2—Cd1—N1	72.0 (2)
C26—C21—C22—C23	0.4 (5)	C1—O2—Cd1—N5	−65.1 (2)
C20—C21—C22—C23	178.7 (3)	C1—O2—Cd1—O7^iv^	−174.3 (2)
C21—C22—C23—C24	−0.7 (6)	C1—O2—Cd1—O1	−2.48 (18)
C22—C23—C24—C25	0.6 (5)	C1—O1—Cd1—N1	−107.7 (2)
C22—C23—C24—C27	178.9 (3)	C1—O1—Cd1—N5	131.9 (2)
C23—C24—C25—C26	−0.3 (5)	C1—O1—Cd1—O7^iv^	15.3 (3)
C27—C24—C25—C26	−178.5 (3)	C1—O1—Cd1—O2	2.53 (18)
C24—C25—C26—C21	0.0 (6)	O1—C1—Cd1—N1	72.2 (2)
C22—C21—C26—C25	0.0 (5)	O2—C1—Cd1—N1	−112.22 (19)
C20—C21—C26—C25	−178.3 (3)	C2—C1—Cd1—N1	−76.3 (16)
C25—C24—C27—N4	−68.2 (4)	O1—C1—Cd1—N5	−50.5 (2)
C23—C24—C27—N4	113.5 (4)	O2—C1—Cd1—N5	125.00 (19)
N3—C29—C30—N4	−0.1 (4)	C2—C1—Cd1—N5	160.9 (16)
N5—C32—C33—N6	0.1 (4)	O1—C1—Cd1—O7^iv^	−169.35 (18)
N6—C34—C35—C37	−20.1 (5)	O2—C1—Cd1—O7^iv^	6.2 (2)
N6—C34—C35—C36	161.8 (3)	C2—C1—Cd1—O7^iv^	42.1 (16)
C37—C35—C36—C37^i^	0.0 (6)	O1—C1—Cd1—O2	−175.5 (3)
C34—C35—C36—C37^i^	178.2 (3)	C2—C1—Cd1—O2	35.9 (15)
C43^ii^—C44—C42—C43	−0.7 (8)	O2—C1—Cd1—O1	175.5 (3)
C43^ii^—C44—C42—C41	177.7 (4)	C2—C1—Cd1—O1	−148.5 (17)
C44—C42—C43—C44^ii^	0.7 (8)	C28—N3—Cd2—N7	−74.1 (3)
C41—C42—C43—C44^ii^	−177.7 (4)	C29—N3—Cd2—N7	120.3 (3)
C36—C35—C37—C36^i^	0.0 (6)	C28—N3—Cd2—O4^iii^	154.3 (2)
C34—C35—C37—C36^i^	−178.1 (4)	C29—N3—Cd2—O4^iii^	−11.4 (3)
C44—C42—C41—N8	58.7 (5)	C28—N3—Cd2—O5	62.8 (3)
C43—C42—C41—N8	−122.9 (4)	C29—N3—Cd2—O5	−102.8 (3)
N7—C39—C40—N8	0.0 (4)	C28—N3—Cd2—O6	11.4 (3)
N2—C17—N1—C18	0.2 (4)	C29—N3—Cd2—O6	−154.3 (3)
N2—C17—N1—Cd1	168.7 (2)	C28—N3—Cd2—C9	36.9 (3)
C19—C18—N1—C17	−0.1 (4)	C29—N3—Cd2—C9	−128.8 (3)
C19—C18—N1—Cd1	−167.8 (2)	C38—N7—Cd2—N3	−46.5 (3)
N1—C17—N2—C19	−0.1 (4)	C39—N7—Cd2—N3	118.9 (3)
N1—C17—N2—C20	178.1 (3)	C38—N7—Cd2—O4^iii^	84.5 (3)
C18—C19—N2—C17	0.1 (4)	C39—N7—Cd2—O4^iii^	−110.1 (3)
C18—C19—N2—C20	−178.3 (3)	C38—N7—Cd2—O5	−177.6 (2)
C21—C20—N2—C17	10.4 (5)	C39—N7—Cd2—O5	−12.2 (3)
C21—C20—N2—C19	−171.6 (3)	C38—N7—Cd2—O6	−130.2 (3)
N4—C28—N3—C29	0.1 (4)	C39—N7—Cd2—O6	35.1 (3)
N4—C28—N3—Cd2	−168.33 (19)	C38—N7—Cd2—C9	−151.5 (3)
C30—C29—N3—C28	0.0 (4)	C39—N7—Cd2—C9	13.9 (3)
C30—C29—N3—Cd2	167.7 (2)	C9—O5—Cd2—N3	−71.0 (2)
N3—C28—N4—C30	−0.2 (4)	C9—O5—Cd2—N7	66.9 (2)
N3—C28—N4—C27	−177.9 (3)	C9—O5—Cd2—O4^iii^	179.39 (19)
C29—C30—N4—C28	0.2 (4)	C9—O5—Cd2—O6	2.24 (17)
C29—C30—N4—C27	178.0 (3)	C9—O6—Cd2—N3	109.3 (2)
C24—C27—N4—C28	−9.2 (5)	C9—O6—Cd2—N7	−129.7 (2)
C24—C27—N4—C30	173.5 (3)	C9—O6—Cd2—O4^iii^	−6.8 (3)
N6—C31—N5—C32	−0.4 (4)	C9—O6—Cd2—O5	−2.28 (18)
N6—C31—N5—Cd1	−165.9 (2)	O6—C9—Cd2—N3	−70.66 (19)
C33—C32—N5—C31	0.2 (4)	O5—C9—Cd2—N3	113.36 (19)
C33—C32—N5—Cd1	165.2 (2)	C10—C9—Cd2—N3	83.7 (17)
N5—C31—N6—C33	0.5 (4)	O6—C9—Cd2—N7	52.9 (2)
N5—C31—N6—C34	178.0 (3)	O5—C9—Cd2—N7	−123.11 (18)
C32—C33—N6—C31	−0.3 (4)	C10—C9—Cd2—N7	−152.8 (17)
C32—C33—N6—C34	−177.9 (3)	O6—C9—Cd2—O4^iii^	175.31 (18)
C35—C34—N6—C31	108.9 (4)	O5—C9—Cd2—O4^iii^	−0.7 (2)
C35—C34—N6—C33	−74.0 (4)	C10—C9—Cd2—O4^iii^	−30.3 (18)
N8—C38—N7—C39	0.3 (4)	O6—C9—Cd2—O5	176.0 (3)
N8—C38—N7—Cd2	168.1 (2)	C10—C9—Cd2—O5	−29.7 (17)
C40—C39—N7—C38	−0.1 (4)	O5—C9—Cd2—O6	−176.0 (3)
C40—C39—N7—Cd2	−168.0 (2)	C10—C9—Cd2—O6	154.3 (19)

Symmetry codes: (i) −*x*+2, −*y*+2, −*z*; (ii) −*x*+1, −*y*−1, −*z*+2; (iii) −*x*+1, −*y*, −*z*+1; (iv) −*x*+2, −*y*+1, −*z*+1.
